# Editorial

**Published:** 1868-03

**Authors:** 


### Editorial.

Annual Meeting of the Illinois State Medical Society.—The next regular Annual Meeting of the Illinois State Medical Society will be held in the City of Quincy, on the *Third Tuesday* in May, 1868.

Summer Course in Chicago Medical College—The regular Summer Course of Instruction in Chicago Medical College, will commence on Monday, March 9th, and continue until the first of July. The course will be so arranged as to consist of one clinic and two lectures each day: the lectures to consist, in part, of thorough examinations of each member of the class. Those participating in this course, will be as follows:

*Clinical Medicine*—Prof. N. S. Davis,
*Clinical Surgery*—Prof. E. Andrews,
*Obstetrics and Diseases of Women*—Dr. E. O. F. Roler,
*Physiology and Histology*—Prof. D. J. Nelson,
*Anatomy, Descriptive*—Prof. J. S. Jewell,
*Operations in Surgery*—Prof. R. N. Isham,
*Materia Medica*—Prof. M. O. Heydock,
*Physical Diagnosis*—Dr. S. A. McWilliams,
*Ophthalmoscope and Ophthalmology*—Prof. E. Andrews.
*Organic Chemistry applied to the Study of Special Pathology*—Prof. N. S. Davis.

The above course of instruction is free to all the regular matriculants of the College.

Valedictory Lecture.—We have received a manuscript copy of Dr. J. S. Hildreth's closing lecture to his clinical course of instruction on Diseases of the Eye and Ear, in the County Hospital. It was a lecture replete with earnest thought, and judicious counsel to his class; but it was received too late for insertion in the present number of the Examiner.

Explanation.—We have received a number of valuable books, the notices of which were crowded out by the length of the article relating to Dr. Bozeman's speculum. Among these works, is a large and important work by Stellway, on Diseases of the Eye, from the publishing house of Wm. Wood & Co., of New York. For sale by W. B. Keen & Co., Chicago.

---

PHYSICIAN'S DAILY POCKET RECORD.—We have received from the author, Dr. S. W. Butler, of Philadelphia, an excellent copy of this work. It possesses all the conveniences afforded by any of the other "Pocket Memoranda" or "Physicians' Visiting Lists," and some that are not found in the others. We commend it to the patronage of the profession.

---

DR. BOZEMAN'S SPECULUM.—Among the selected articles in this number of the EXAMINER, is one from the *New York Medical Record*, giving an account of a new and improved vaginal speculum. It has been used by its author in several operations for vesico-vaginal fistula, and with the most satisfactory results.

---

### MEDICAL ADVERTISING.

*To the Editor of the Medical Examiner*—SIR:—Wishing to correct any misunderstanding which may occur from the article in your February No., entitled "Give an Inch and an Ell is Taken," we respectfully request the publication of the following:

Having decided to devote ourselves exclusively to the treatment of diseases of the genito-urinary organs, we called on several members of the profession and faculties of the medical schools of this city, explaining to them our business, and, also, our intention of bringing it before the public through the medium of the *papers*, by the insertion of a card, as well as by the ordinary business card, and with this express understanding obtained all the names we used as references, some of whom have since requested us to suspend the further publication of their names in the newspapers; but none have forbidden their use on our business card. The appearance of our card in the "Medical Column," as referred to, was contrary to our intention or desire, but, at the time, beyond our control.

Respectfully, DRS. BROWN & HIGGINS.

*Feb. 15, 1868.* *119 Clark St., Chicago.*

### Mortality Report for the Month of January:—

### CAUSES OF DEATH.

| | | | | | |
|---|---|---|---|---|---|
| Accident, concussion of brain | 1 | Eclampsia | 1 | Meningitis | 3 |
| Accident, poison | 1 | Encephalites | 2 | " cereb.-spinal | 4 |
| Accident, burns | 2 | Enteritis | 2 | " tuberculous | 1 |
| Accident, fall | 1 | Enteritis, chronic | 1 | Myelitis | 2 |
| Accident, suffocation | 1 | Encephalus, general | 1 | Nutrition, impaired | 1 |
| Anæmia | 1 | Fever, intermittent | 1 | Nephritis | 1 |
| Angina | 1 | Fever, puerperal | 2 | Old Age | 4 |
| Apoplexy | 3 | Fever, remittent | 1 | Œsophagus, stricture | 1 |
| Bowels, inflammation | 5 | Fever, scarlet | 7 | Occipital bone, depression of | 1 |
| Brain, congestion of | 5 | Fever, typhoid | 14 | Paralysis | 2 |
| Brain, dropsy of | 1 | Gastritis | 3 | Pericarditis | 1 |
| Brain, disease of | 1 | Gangrene | 1 | Peritonitis | 3 |
| Brain, inflammation | 2 | Gangrene, leg | 1 | Phthisis pulmonalisis | 38 |
| Bright's disease | 2 | Gout, rheumatic | 1 | Poisoned | 1 |
| Bronchitis | 9 | Glands of neck, inflammation of | 1 | Pleurisy | 2 |
| Bronchitis, capillary | 2 | Heart, disease of | 13 | Pneumonia | 49 |
| Cancer | 2 | Hemiplegia | 1 | Small-pox | 39 |
| Cancer of breast | 1 | Hepatitis | 1 | Spine, disease of | 1 |
| Cancer of uterus, | 2 | Hernia | 1 | Stomach, disease of | 1 |
| Canker sore mouth | 1 | Hydrocephalus | 9 | Suicide | 3 |
| Convulsions | 51 | Indigestion | 1 | Syncope | 1 |
| Croup | 8 | Inanition | 2 | Syphilis | 2 |
| Cyanosis | 1 | Intemperance | 4 | Tabes mesenterica | 7 |
| Cyanche trachealis | 1 | Intestines, perforation | 1 | Teething | 6 |
| Cyanche maligna | 1 | Jaundice | 2 | Throat, malformation | 1 |
| Cerebellum, disease of | 1 | Kidneys, inflammation | 2 | Uræmia | 1 |
| Debility | 4 | Laryngitis | 5 | Uterus, hemorrhage of | 1 |
| Diarrhœa | 3 | Liver, fatty enlargem't | 1 | Varioloid | 1 |
| Diphtheria | 21 | Liver, congestion of | 1 | Whooping-cough | 3 |
| Dropsy | 1 | Lungs, congestion of | 9 | Unknown | 3 |
| Dropsy, abdomen | 1 | Lungs, hemorrhage of | 1 | Still and Premature births | 39 |
| Dysentery | 2 | Measles | 21 | | |

Total 438

Deaths in January, 1868, 438 | Deaths in January, 1867, 299 | Increase, 139
Deaths in December, 1867, 409 | Increase, 29

### AGES.

| | | | | | |
|---|---|---|---|---|---|
| Under 5 | 252 | 40 to 50 | 28 | 90 to 100 | 1 |
| 5 to 10 | 20 | 50 to 60 | 7 | 100 to 110 | 0 |
| 10 to 20 | 14 | 60 to 70 | 17 | Unknown | 0 |
| 20 to 30 | 38 | 70 to 80 | 11 | | |
| 30 to 40 | 47 | 80 to 90 | 3 | Total | 438 |

Males, 224 | Females, 214 | Total, 438

### NATIVITY.

| | | | | | |
|---|---|---|---|---|---|
| Chicago | 191 | Germany | 48 | Russia | 1 |
| Other parts U. S. | 101 | Holland | 2 | Scotland | 4 |
| Australia | 1 | Ireland | 46 | Sandwich Islands | 1 |
| Belgium | 0 | Italy | 0 | Sweden | 2 |
| Bohemia | 5 | Norway | 14 | Switzerland | 1 |
| Canada | 7 | Nova Scotia | 0 | Wales | 0 |
| Denmark | 0 | Prussia | 4 | Unknown | 2 |
| England | 7 | Poland | 0 | | |
| France | 3 | | | Total | 438 |

### MORTALITY BY WARDS FOR THE MONTH.

| Ward. | Mortality. | Pop. in 1866. | One death in |
|---|---|---|---|
| 1 | 8 | 9,668 | 1,208 1-2 |
| 2 | 16 | 12,985 | 811 9-16 |
| 3 | 24 | 15,738 | 655 9-12 |
| 4 | 32 | 10,884 | 380 1-8 |
| 5 | 18 | 9,610 | 536 1-9 |
| 6 | 27 | 10,680 | 391 7-9 |
| 7 | 35 | 18,755 | 538 5-35 |
| 8 | 23 | 10,429 | 453 10-23 |
| 9 | 33 | 13,940 | 422 14-33 |
| 10 | 18 | 11,416 | 634 2-9 |
| 11 | 36 | 12,924 | 359 |
| 12 | 32 | 12,695 | 396 2-3 |
| 13 | 13 | 8,188 | 629 11-13 |
| 14 | 27 | 12,108 | 448 12-27 |
| 15 | 39 | 15,766 | 404 1-4 |
| 16 | 26 | 14,912 | 573 7-13 |
| County hosp., | 7 | | |
| Armory, | 1 | | |
| Bridewell, | 1 | | |
| Home of the Friendl's, | 0 | | |
| Marine hos., | 2 | | |
| Mercy hos., | 2 | | |
| St. Luke's hos. | 3 | | |
| St. Jose. asyl. | 16 | | |
| Lake Hosp., | 5 | | |
| Total, | 401 | | |

### DEATHS BY SMALL-POX.

| | Jan., 1868. | Week ending Feb. 1, 1868. |
|---|---|---|
| 3d Ward | 1 | 0 |
| 4th Ward | 5 | 0 |
| 5th Ward | 6 | 5 |
| 6th Ward | 3 | 1 |
| 7th Ward | 6 | 3 |
| 8th Ward | 5 | 0 |
| 9th Ward | 1 | 1 |
| 10th Ward | 0 | 1 |
| 11th Ward | 3 | 2 |
| 12th Ward | 3 | 0 |
| 14th Ward | 1 | 0 |
| 15th Ward | 1 | 1 |
| 16th Ward | 1 | 0 |
| Lake Hospital, | 3 | 1 |
| Total | 39 | 15 |

TO PHYSICIANS.—His attempts to extend a more advanced knowledge of his specialty to physicians already in practice having been so favorably commented upon by those of the profession who have attended the previous courses, and by the medical press. **PROF. HORATIO R. STORER,** Will deliver his third private course of twelve Lectures upon the

**TREATMENT OF THE SURGICAL DISEASES OF WOMEN,**

During the first fortnight of June, at his rooms in Boston.

Fee $50; and Diploma required to be shown. Certificates of attendance upon the courses already completed have been issued to the following gentlemen: Drs. J. B. Walker, Union, Me.; Alexander J. Stone, Augusta, Me.; Daniel Mann, Pelham, N.H.; Augustus Harris, Colebrook, N.H; J. W. Parsons, Portsmouth, N.H.; E. F. Upham, West Randolph, Vt.; G. E. Bullard, Blackstone, Mass.; J. A. McDonough, Boston, Mass.; J. G. Pinkham, Cambridge, Mass.; James Coolidge, Athol, Mass.; Thomas G. Potter, Providence, R.I.; C. M. Carleton, Norwich, Conn.; I. Farrar, Hartford, Conn.; M. C. Talbott, Warren, Pa.; H. Gerould, Erie, Pa.; W. W. Bancroft, Granville, Ohio; A. I. Beach, Bellville, Ohio; Henry E. Paine, Dixon, Ill.; W. L. Wells, Howall, Mich.; and W. A. I. Case, Hamilton, C.W.

Hotel Pelham, Boston, January, 1868. feb3t.

MONEY RECEIPTS TO FEB. 24TH.—Drs. V. L. [illegible]ibut, $3; W. Fleming, 3; J. S. Sherman, 3; Theodore Hoffman, 3; J. Haller, 3; M. Shephard, 3; W. H. Baxter, 5; H. W. Pickett, 3; J. Good, 3; E. Young, 6; L. Corey, 3; M. Parker, 3; G. W. P. Hadder, 3; W. A. Barstow, 3; H. A. Stokes, 3; John Ten Brook, 5; W. M. Landon, 6; H. C. Lester, 3.

---

NUMBER OF EMIGRANTS WHO HAVE ARRIVED IN NEW YORK DURING PAST YEAR.—Two hundred and thirty-three thousand four hundred and eighteen.

---

THE CHOLERA has again made its appearance in India, as was expected, indeed, as the result of the pilgrimage of the past year. Apropos of the cholera: a French writer claims as a prominent cause of the pestilence the disappearance of a variety of crocodile, called the gavial. This animal formerly destroyed the bodies thrown into the Ganges, in accordance with the religious customs of India. But since the soldiers have been in the habit of waging war upon it, the gavial has been nearly exterminated. In the absence of its scavenger work, the bodies decay and produce the cholera; at least so claims this writer, who asks for a government protection of this important reptile.

---

### AMERICAN MEDICAL ASSOCIATION—PRIZE ESSAYS FOR 1868.

The American Medical Association offers two prizes of *One Hundred Dollars* each, for the best two original essays upon subjects of professional interest; the Committee reserving the right to reject all, unless deemed fully worthy.

Competitors for these prizes must forward their essays to Dr. Charles Woodward, Cincinnati, Ohio, free of expense, on or before the 1st of April, 1868.

Each essay must be accompanied by a sealed note containing the author's name and address, and on this sealed packet must be inscribed some sentiment, motto, or device, corresponding to a like sentiment, motto, or device on the essay.

CHARLES WOODWARD, Chairman,<br>

W. W. DAWSON,<br>

E. B. STEVENS,<br>

ROBERTS BARTHOLOW,<br>

P. S. CONNOR,<br>

Committee.

Medical journals throughout the country are requested to copy.

